# The effects of hemoglobin levels and their interactions with cigarette smoking on survival in nasopharyngeal carcinoma patients

**DOI:** 10.1002/cam4.647

**Published:** 2016-01-28

**Authors:** Qi Zeng, Lu‐Jun Shen, Sheng Li, Ling Chen, Xiang Guo, Chao‐Nan Qian, Pei‐Hong Wu

**Affiliations:** ^1^ State Key Laboratory of Oncology in South ChinaCollaborative Innovation Center for Cancer MedicineGuangzhou510060China; ^2^ Department of Medical Imaging and Interventional OncologySun Yat‐sen University Cancer CenterGuangzhouChina; ^3^ Department of Statistical Analysis and Medical RecordsThe Fifth Affiliated Hospital of Sun Yat‐sen UniversityZhuhaiChina; ^4^ Department of Nasopharyngeal CarcinomaSun Yat‐sen University Cancer CenterGuangzhouChina

**Keywords:** Hemoglobin, interaction, nasopharyngeal carcinoma, radiotherapy, smoking

## Abstract

There is very little published information regarding the prognostic value of hemoglobin (Hb) levels combined with smoking on the survival of patients with nasopharyngeal carcinoma (NPC), and the interactions between them remain unclear. A total of 2440 NPC patients were confirmed, and multivariate analysis was performed to identify valuable prognostic Hb levels in the entire population and in the cohort of smokers. The survival differences were compared using log‐rank tests. The multiplicative and additive interactions were assessed using Cox regression and a Microsoft Word Excel spreadsheet. Postradiotherapy (RT) Hb was an independent prognostic factor for overall survival (OS) (HR = 0.797; *P *=* *0.006), failure‐free survival (FFS) (HR=0.811; *P *=* *0.010), and loco‐regional failure‐free survival (LR‐FFS) (HR = 0.725; *P *=* *0.000). In the cohort of smokers, pack‐years was also an independent predictor of OS (HR = 0.673; *P *<* *0.001) and FFS (HR = 0.681; *P *<* *0.001), LR‐FFS (HR = 0.663; *P *=* *0.001). A significant positive additive effect was found for the interaction between low post‐RT Hb and high SI on OS, with RERI = 5.616, AP = 0.665, and S = 4.078. Stratified analyses demonstrated that heavy smokers with low post‐RT Hb had HRs of 2.295 (*P *<* *0.001) for death, 2.222 (*P *<* *0.001) for disease failure, and 2.267 (*P *<* *0.001) loco‐regional recurrence compared with light smokers with high post‐RT Hb levels, and post‐RT Hb level is an important predictor of survival in patients with NPC. The positive interaction between post‐RT Hb level and pack‐years contributes to the elevated risk of poor survival. Oncologists should devote particular attention to heavy smokers with low post‐RT Hb levels in the future.

## Background

Numerous studies have demonstrated that low hemoglobin (Hb) is a strong prognostic indicator of poor disease control and survival in patients with head and neck squamous cell carcinomas (HNSCC) 1, 2, 3. Nasopharyngeal carcinoma (NPC) is a distinct form of HNSCC in terms of epidemiology, pathology, and response to treatment 4, 5, 6. Chua and his colleagues reported low mid‐radiation Hb levels to be an independent predictor of local disease recurrence in NPC patients 7, which is similar to the findings of previous studies on HNSCC. It is generally assumed that poor tumor control in patients with low Hb levels is primarily a consequence of tumor hypoxia 8, 9. Additionally, previous studies 10, 11 have shown that cigarette smoking is a poor prognostic factor for NPC patients. Smoking exacerbates tissue hypoxia and can lead to smoking‐induced tissue hypoxia in healthy smokers 12. Although sufficient supporting data have revealed that low Hb levels and smoking are related to tissue hypoxia, no studies address the prognostic value of Hb levels combined with cigarette smoking, and the interactions between them remain unclear.

In this study, we used data obtained from a large database of NPC patients in our institution to validate the effects of hemoglobin levels and their interactions with cigarette smoking on survival in NPC patients.

## Materials and Methods

### Patient population

The patients selected in this study received treatment at our institution between January 2001 and January 2005. The study was approved by the institutional reviewed board and the hospital ethics committee. This was a retrospective analysis, and therefore we were granted a waiver of individual informed consent. The data were collected by trained interviewers and analyzed anonymously. The inclusion criteria for this study were the following: (1) histologically confirmed nonkeratinizing NPC (World Health Organization type II or III); (2) KPS (Karnofsky performance scale) score ≥80; (3) newly diagnosed patients without evidence of systemic metastasis; and (4) completion of the scheduled total radiotherapy dose. The exclusion criteria were the following: (1) follow‐up period less than 5 years; (2) lack of complete Hb recorded; and (3) lack of a record of smoking status (including the patients whose records differed from those of physicians and nurses). Medical records were reviewed to collect data on the patients' clinical features and smoking histories, including age, gender, cancer stage based on the sixth edition of the Union for International Cancer Control/American Joint Committee on Cancer (UICC/AJCC) 13, radiotherapy dose, treatment group [radiotherapy or combined chemo‐radiotherapy (CRT)], BMI [defined as preradiotherapy (pre‐RT) weight (kg) divided by the square of height (meter)], smoking status at diagnosis, number of cigarettes smoked per day, number of years of smoking, and number of years since cessation. Patients were divided into three categories 10, 11: (1) never‐smokers referred to patients who had never smoked; (2) ex‐smokers referred to former smokers who had stopped smoking for 1 year or more before treatment; and (3) smokers referred to smokers who did so until the day of hospitalization or who had stopped smoking but for less than 1 year. Pack‐years was calculated by multiplying the number of packs (1 pack has 20 cigarettes) of cigarettes smoked per day by the number of years a person had smoked.

Hemoglobin was measured prior to or after radiotherapy, and patients were also serially monitored once every week during the course of treatment. If the result was abnormal, then Hb was examined two or three times per week. Preradiotherapy Hb levels referred to values measured 1 week prior to the start of radiotherapy. Mid‐radiotherapy (mid‐RT) Hb levels referred to the mean of all Hb values during the course of radiotherapy. Postradiotherapy (post‐RT) Hb levels referred to the Hb values at the last week of RT.

### Treatment

The radiotherapy techniques were two‐dimensional conventional radiotherapy, three‐dimensional conformal radiotherapy (3DRT), and intensity‐modulated radiotherapy (IMRT). These details were previously described by Shen et al. 14. Briefly, conventional radiation therapy was performed with 2 Gy per fraction with five daily fractions per week up to a total dose of 68–78 Gy. For 3DCRT, the dose of 66–72 Gy was prescribed for gross tumors in the nasopharynx (GTVnx), and 60–70 Gy was prescribed for metastatic lymph node involvement (GTVnd). For IMRT, the prescription doses were 68 Gy to GTVnx or 60–64 Gy to GTVnd. Combined modality therapy for most locoregionally advanced NPC entailed induction chemotherapy followed by RT (IC + RT), concurrent chemoradiotherapy (CCRT) plus or not plus adjuvant chemotherapy (AC). The IC or AC regimen was mainly cisplatin plus fluorouracil (5‐Fu), with cisplatin (70–100 mg/m^2^) given on day 1 and 5‐fluorouracil (500–750 mg/m^2^) on days 1–5, repeated every 3–4 weeks, for 2–3 cycles. The CCRT regimen was mainly cisplatin alone, with 30–40 mg/m^2^ cisplatin given intravenously weekly for 5–7 cycles or 80–100 mg/m^2^ given intravenously every 3 weeks for three cycles.

### Follow‐up and endpoints

The last follow‐up occurred in August 2011. The primary endpoint was overall survival (OS), and the secondary endpoints were failure‐free survival (FFS), locoregional failure‐free survival (LR‐FFS), and distant failure‐free survival (D‐FFS). OS was defined as the length of time from the date of beginning therapy to the date of death from any cause. FFS was defined as the time from the date of beginning therapy to the date of treatment failure or death from any cause. LR‐FFS was defined as the time to first recurrence at the nasopharyngeal region and/or in the cervical region after radiotherapy, not including salvage procedures. D‐FFS was defined as the time from the date of beginning therapy to the first distant failure.

### Statistical analysis

Statistical analyses were performed using SPSS 19.0.0 (SPSS Inc., Chicago, IL, USA). Correlation analysis was conducted with the Spearman's correlation test. Partial correlation analysis was performed between a given two variables while the effects of other variables were controlled for. Receiver operating characteristic (ROC) curve analyses were used to select the cutoff points for Hb levels and pack‐years. The median follow‐up time was calculated using the reverse Kaplan–Meier (KM) estimator 15. The survival curves were evaluated using the Kaplan–Meier method, and log‐rank tests were performed to compare the survival differences. A two‐tailed *P* value of less than or equal to 0.05 was considered statistically significant. A multivariate Cox proportional hazards analysis for OS, FFS, LR‐FFS, and D‐FFS, with stepwise backward elimination of variables at *P *>* *0.1, was performed. The multiplicative interactions between Hb levels and cigarette smoking were assessed using Cox regression. The additive interactions were assessed using an Excel spreadsheet that was developed by Tomas Andersson et al. 16. Rothman presented three measures of additive interaction: RERI, the relative excess risk due to interaction; AP, the attributable proportion due to interaction; and S, the synergy index 17. If there is no biological interaction, RERI and AP are equal to 0 and S is equal to 1 18.

## Results

### Demographics, patterns of treatment failure, and survival

As shown in Table 1 and Fig. S1, a total of 2440 NPC patients were included in this study, with a median age of 46 years (range, 11–78 years). The ratio of males to females was 3.18:1, with 1856 males and 584 females. The UICC/AJCC clinical stage distribution groupings were the following: stage I, 127 (5.2%); stage IIa, 25 (1.0%); stage IIb, 832 (34.1%); stage III, 984 (40.3%); stage IVa, 383 (15.7%); and stage IVb, 89 (3.6%). Overall, 1090 (44.7%) patients were treated with radiotherapy (RT) alone and 1350 (55.3%) received combined CRT. The median follow‐up was 94.0 months (95% CI: 92.9–95.2 months). Five hundred sixty‐seven (23.2%) patients developed locoregional relapse, 165 (6.8%) developed distant metastases, and 756 (31.0%) died. The 5‐year survival rates were as follows: OS, 74.8%; FFS, 73.7%; LR‐FFS, 80.6%; and D‐FFS, 94.1%.

**Table 1:** Patient and tumor characteristics in the entire patient cohort

Characteristics	
Age (year) media (range)	46 (11–78)
Gender (%)	
Male	1856 (76.1)
Female	584 (23.9)
BMI (kg/m^2^), media (range)	22.41 (13.61–39.06)
Smoking status (%)	
Never‐smokers	1272 (52.1)
Ex‐smokers + smokers	1168 (47.9)
Pre‐RT Hb (g/dL) media (range)	145 (76–211)
Mid‐RT Hb (g/dL) media (range)	131 (65–173.4)
Post‐RT Hb (g/dL) media (range)	128 (64–178)
Clinical stage (%)	
I–II	984 (40.3)
III–IV	1456 (59.7)
T stage (%)	
T1‐2	1420 (58.2)
T3‐4	1020 (41.8)
N stage (%)	
N0‐1	1613 (66.1)
N2‐3	827 (33.9)
Treatment group (%)	
RT	1090 (44.7)
CRT	1350 (55.3)
RT dose (Gy), media (range)	70 (60–87)

BMI, body mass index; Hb, hemoglobin; RT, radiotherapy; CRT, combined chemo‐radiotherapy.

### Hemoglobin levels, smoking status, and correlation analyses

The median pre‐RT, mid‐RT, and post‐RT Hb levels were 14.41 g/dL (range, 7.6–21.1 g/dL), 12.98 g/dL (range, 6.5–17.34 g/dL), and 12.74 g/dL (range, 6.4–17.8 g/dL), respectively. The mean decrease between pre‐RT and post‐RT Hb levels was 2.01 g/dL (range, 0.1–9.8 g/dL). Most patients, 85.7%, had a measured decrease and 39.7% of patients had a decrease of ≥2 g/dL. Thirty‐nine patients had the same pre‐RT and post‐RT Hb levels, and 12.7% of patients showed increases. The proportions of never‐smokers, ex‐smokers, and smokers were 52.1% (1272/2440), 8.5% (208/2440), and 39.3% (960/2440), respectively. In smokers and ex‐smokers, the vast majority of patients (>99%) were male. Compared with never‐smokers, the mean pre‐RT (14.09 g/dL vs. 14.77 g/dL, *P *<* *0.005), mid‐RT (12.77 g/dL vs. 13.20 g/dL, *P *<* *0.005), and post‐RT (12.56 g/dL vs. 12.91 g/dL, *P *<* *0.005) Hb levels were significantly higher among smokers and ex‐smokers, but there were no significant differences between ex‐smokers and smokers (*P *>* *0.05).

Correlation analyses were performed between Hb levels and smoking status, and smoking status was slightly correlated with pre‐RT (R = 0.206, *P *<* *0.001), mid‐RT (R = 0.138, *P *<* *0.001), and post‐RT (R = 0.099, *P *<* *0.001) Hb levels. However, the correlations were not significant (*P *>* *0.05) after age, gender, and clinical stage were controlled for. Among the smokers (excluding four females), after age and clinical stage were controlled for, our analyses also showed that there were no correlations between pack‐years and pre‐RT (R = 0.042, *P *=* *0.193), mid‐RT (R = ‐0.003, *P *=* *0.926), or post‐RT (R = ‐0.009, *P *=* *0.792) Hb levels.

### Determining cutoff points for hemoglobin levels and pack‐years

Because OS was the primary endpoint in this study, the cutoff point for OS was selected as the optimal value using ROC curve analysis. The results indicated that the pre‐RT Hb cutoff was 14.25 g/dL (the sensitivity was 59.0%, and the specificity was 45.9%) with an area of 0.522 (95% CI, 0.497–0.546; *P *=* *0.088). Similarly, the mid‐RT Hb cutoff was 13.06 g/dL (sensitivity: 55.0%, specificity: 54.6%) with an area of 0.556 (95% CI, 0.531–0.580; *P *<* *0.001), and the post‐RT Hb cutoff was 12.45 g/dL (sensitivity: 62.2%, specificity: 47.9%) with an area of 0.564 (95% CI, 0.540–0.589; *P *<* *0.001). In addition, the pack‐years cutoff was 27.5 (sensitivity: 49.6%, specificity: 64.6%) with an area of 0.593 (95% CI, 0.555–0.631; *P *<* *0.001). Based on these established cutoff points, the Hb levels were further divided into high and low, and patients were divided into heavy and light smokers.

### Impact of Hb levels and smoking status on survival

**Figure 1:**
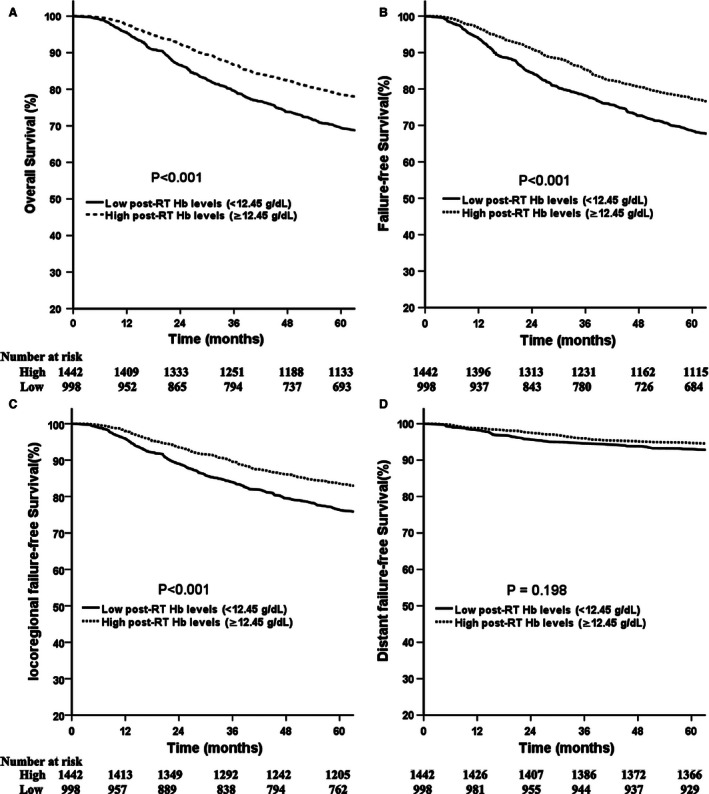
Comparison of survival between patients with high and low post‐RT Hb levels.

Compared with patients with low post‐RT Hb levels, patients with high levels had significantly better 5‐year OS (78.6% vs. 69.4%, *P *<* *0.001; Fig. 1A), FFS (77.3% vs. 68.5%, *P *<* *0.001; Fig. 1B), and LR‐FFS (83.6% vs. 76.4%, *P *<* *0.001; Fig. 1C). No significant benefit was observed for D‐FFS (94.7% vs. 93.1%, *P *=* *0.198; Fig. 1D) between the two groups. Similarly, patients with high mid‐RT Hb levels had a significantly better 5‐year OS, FFS, and LR‐FFS compared with patients with low levels (Fig. S2). Additionally, the 5‐year OS and FFS for patients with high pre‐RT Hb levels were significantly better compared with the rates for patients with low pre‐RT Hb levels (Fig. S3)

Ex‐smokers and smokers were considered as a single group because they have similar survival rates. The 5‐year OS, FFS, LR‐FFS, and D‐FFS were 79.0% vs. 70.3% (*P *<* *0.001), 77.8% vs. 69.3% (*P *<* *0.001), 83.9% vs. 77.1% (*P *<* *0.001), 94.7% vs. 93.3% (*P *=* *0.246) between nonsmokers and smokers (including ex‐smokers).

### Multivariate analyses of potentially important prognostic factors and the measurement of the interactions

In the entire population, the potentially important prognostic factors that were considered in the multivariate analyses were age (continuous variable), gender (female vs. male), T stage (T1‐2 vs. T3‐4), N stage (N0‐1 vs. N2‐3), treatment group (RT vs. CRT), BMI (continuous variable), smoking status (never‐smokers vs. ex‐smokers plus smokers), and pre‐RT (≥ vs. <14.25 g/dL), mid‐RT (≥ vs. <13.06 g/dL), and post‐RT Hb levels (≥ vs. <12.45 g/dL). (Smoking status) × (Pre‐RT Hb level), (Smoking status) × (mid‐RT Hb level), and (Smoking status) × (post‐RT Hb level) were also included to analyze the multiplicative interactions between Hb levels and cigarette smoking. Table 2 summarizes the multivariate analyses of important prognostic factors on OS, FFS, LR‐FFS, and D‐FFS. The results indicated that high post‐RT Hb levels were associated with OS (HR = 0.797, 95% CI, 0.678‐0.936), FFS (HR = 0.811, 95% CI, 0.692–0.951), and LR‐FFS (HR = 0.725, 95% CI, 0.608–0.865); thus, post‐RT Hb was an independent prognostic factor for OS (*P *=* *0.006), FFS (*P *=* *0.010), and LR‐FFS (*P *<* *0.001). Pre‐RT and mid‐RT Hb levels and smoking status were not independent prognostic factors for survival; thus, these levels were excluded from the analyses of additive interactions. There were also no multiplicative interactions between Hb levels and smoking status in terms of survival.

**Table 2:** Multivariate analyses in the entire population

	OS		FFS		LR‐FFS		D‐FFS	
	HR (95% CI)	*P*	HR (95% CI)	*P*	HR (95% CI)	*P*	HR (95%CI)	*P*
Age	1.032 (1.026–1.039)	<0.001	1.031 (1.025–1.038)	<0.001	1.026 (1.019–1.033)	<0.001	1.013 (1.000–1.026)	0.051
Gender								
Female vs. male	0.677 (0.559–0.820)	<0.001	0.692 (0.573–0.836)	<0.001	0.605 (0.483–0.758)	<0.001	1.007 (0.589–1.721)	0.981
T stage								
T1‐2 vs. T3‐4	0.777 (0.670–0.902)	0.001	0.794 (0.686–0.920)	0.002	0.773 (0.654–0.913)	0.002	1.504 (0.768–1.445)	0.746
N stage								
N0‐1 vs. N2‐3	0.683 (0.586–0.795)	<0.001	0.691 (0.594–0.803)	<0.001	0.717 (0.604–0.851)	<0.001	0.805 (0.584–1.108)	0.183
Treatment group								
RT vs. CRT	0.833 (0.704–0.986)	0.033	0.814 (0.690–0.962)	0.016	0.929 (0.763–1.131)	0.464	0.422 (0.298–0.598)	<0.001
BMI	0.946 (0.923–0.968)	<0.001	0.948 (0.926–0.970)	<0.001	0.964 (0.938–0.990)	0.008	0.936 (0.891–0.983)	0.009
Smoking status	0.874 (0.674–1.132)	0.306	0.878 (0.681–1.134)	0.320	0.875 (0.649–1.180)	0.383	0.820 (0.516–1.304)	0.402
Pre‐RT Hb	0.916 (0.776–1.082)	0.304	0.923 (0.783–1.088)	0.340	0.907 (0.746–1.102)	0.325	0.924 (0.575–1.485)	0.745
Mid‐RT Hb	1.074 (0.821–1.406)	0.601	1.041 (0.798–1.357)	0.768	1.135 (0.846–1.522)	0.400	0.822 (0.551–1.228)	0.339
Post‐RT Hb	0.797 (0.678–0.936)	0.006	0.811 (0.692–0.951)	0.010	0.725 (0.608–0.865)	<0.001	1.186(0.856–1.644)	0.306
Smoking status × Pre‐RT Hb	1.104 (0.788–1.546)	0.564	1.129 (0.810–1.574)	0.474	1.016 (0.684–1.509)	0.938	1.081 (0.657–1.778)	0.760
Smoking status × Mid‐RT Hb	0.904 (0.740–1.105)	0.324	0.899 (0.738–1.095)	0.290	0.837 (0.662–1.059)	0.138	1.023 (0.448–2.336)	0.957
Smoking status × Post‐RT Hb	1.234 (0.918–1.658)	0.163	1.272 (0.952–1.698)	0.103	1.305 (0.926–1.838)	0.128	1.220 (0.660–2.255)	0.526

RT, radiotherapy alone; CRT, combined chemo‐radiotherapy; BMI: body mass index; Smoking status, never‐smokers vs. (ex‐smokers plus smokers); Pre‐RT Hb, Hb levels ≥ vs. <14.25 g/dL; Mid‐RT Hb, Hb levels ≥ vs. <13.06 g/dL; Post‐RT, Hb levels ≥ vs. <12.45 g/dL.

In the cohort of smokers, potentially important prognostic factors were T stage (T1–2 vs. T3‐4), N stage (N0‐1 vs. N2‐3), treatment group (RT vs. CRT), BMI (continuous variable), pack‐years (< vs. ≥27.5), post‐RT Hb level (≥ vs. <12.45 g/dL), and pack‐years × post‐RT Hb level. Gender and age were excluded as covariates because there were only four female patients among the smokers, but age was correlated with pack‐years (*R* = 0.542, *P *<* *0.001). As shown in Table 3, post‐RT Hb (≥ vs. <12.45 g/dL) was found to be a significant and independent predictor of OS (HR *R* 0.623, 95% CI, 0.497–0.780; *P *<* *0.001), FFS (HR = 0.630, 95% CI, 0.504–0.787; *P *<* *0.001), and LR‐FFS (HR = 0.555, 95% CI, 0.433–0.711; *P *<* *0.001). Similarly, pack‐years (< vs. ≥27.5) was also an independent predictor of OS (HR=0.673, 95% CI, 0.542–0.835; *P *<* *0.001), FFS (HR = 0.681, 95% CI, 0.550–0.844; *P *<* *0.001), and LR‐FFS (HR = 0.663, 95% CI, 0.519–0.846; *P *=* *0.001). There was no multiplicative interaction between post‐RT Hb level and pack‐years for survival.

**Table 3:** Significant factors for long‐term survival by multivariate analysis in male smokers

Variables	OS		FFS		LR‐FFS		D‐FFS	
	HR (95% CI)	*P*	HR (95% CI)	*P*	HR (95% CI)	*P*	HR (95%CI)	*P*
T stage								
T1‐2 vs. T3‐4	0.790 (0.635–0.982)	0.034	0.787 (0.634–0.976)	0.029	0.805 (0.629–1.030)	0.085	1.032 (0.636–1.673)	0.899
N stage								
N0‐1 vs. N2‐3	0.810 (0.649–1.012)	0.064	0.808 (0.648–1.007)	0.057	0.826 (0.641–1.063)	0.137	1.093 (0.667–1.791)	0.724
BMI (m^2^/kg)	0.959 (0.923–0.995)	0.026	0.960 (0.925–0.996)	0.028	0.976 (0.936–1.018)	0.258	0.927 (0.856–1.004)	0.064
Treatment group								
RT vs. CRT	0.883 (0.683–1.142)	0.343	0.854 (0.662–1.103)	0.227	1.014 (0.759–1.354)	0.924	0.339 (0.189–0.609)	<0.001
SI (pack‐years)								
Light vs.Heavy smokers	0.673 (0.542–0.835)	<0.001	0.681 (0.550–0.844)	<0.001	0.663 (0.519–0.846)	0.001	1.307 (0.617–2.770)	0.484
Hb levels (g/dL)								
High vs. Low Hb	0.623 (0.497–0.780)	<0.001	0.630 (0.504–0.787)	<0.001	0.555 (0.433–0.711)	<0.001	1.189 (0.720–1.963)	0.499

BMI, body mass index; RT, radiotherapy alone; CRT, combined chemo‐radiotherapy; SI, Smoking index; Light vs. Heavy smokers: Smoking index < vs. ≥ 27.5 pack‐years; High vs. Low Hb: post‐RT Hb levels ≥ vs. <12.45 g/dL.

**Figure 2:**
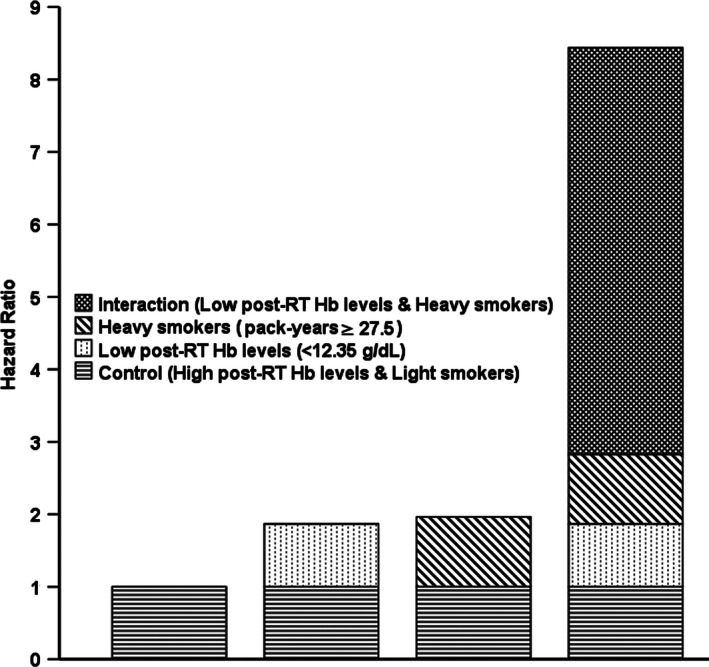
Hazard ratios with contributions from different categories of post‐RT Hb levels and pack‐years. RT: radiotherapy.

In the analysis of additive interactions, after T stage (T1‐2 vs. T3‐4), N stage (N0‐1 vs. N2‐3), treatment group (RT vs. CRT), and BMI (continuous variable) were adjusted for, a significant positive additive interaction effect between low post‐RT Hb level (<12.45 g/dL) and heavy smokers (pack‐years ≥27.5) on OS was found among smokers, with RERI = 5.616 (95% CI, 2.599–8.632), AP = 0.665 (95% CI, 0.523–0.808), and *S* = 4.078 (95% CI, 2.241–7.420), as shown in Fig. 2. These results showed that 66.5% of patient deaths could be attributed to the additive interaction between low post‐RT Hb level (<12.45 g/dL) and heavy smoking (pack‐years ≥27.5).

### Stratified analyses of different categories of smokers

To assess the prognostic impact of different categories of post‐RT Hb level and pack‐years on survival, an additional stratified analysis was conducted (Table 4). After T stage, N stage, treatment group, and BMI were adjusted for, heavy smokers with low post‐RT Hb had HRS of 2.295 (95% CI, 1.684–3.129; *P *<* *0.001) for death, 2.222 (95% CI, 1.635–3.018; *P *<* *0.001) for disease failure, and 2.267 (95% CI, 1.887–3.714; *P *<* *0.001) for locoregional recurrence compared with light smokers with high post‐RT Hb levels. Similarly, heavy smokers with high post‐RT Hb had HRs of 1.615 (95% CI, 1.201–2.170; *P *=* *0.001) for death compared with light smokers with high post‐RT Hb levels. Heavy smokers with low post‐RT Hb had an HR for death of 1.468 (95% CI, 1.067–2.019; *P *=* *0.018) compared with heavy smokers with high post‐RT Hb levels. There were no significant differences in survival between heavy smokers with high post‐RT Hb and light smokers with low post‐RT Hb levels (Table 4).

**Table 4:** Subgroup multivariable analyses in male smokers adjusted for T stage, N stage, treatment group, and BMI

Post‐RT Hb levels	Smoking index	No.	OS		FFS		LR‐FFS	
			HR (95% CI)	*P*	HR (95% CI)	*P*	HR (95% CI)	*P*
High	Light	386	Ref		Ref		Ref	
Low	Heavy	157	2.295 (1.684–3.129)	<0.001	2.222 (1.635–3.018)	<0.001	2.267 (1.887–3.714)	<0.001
High	Light	386	Ref		Ref		Ref	
High	Heavy	228	1.615 (1.201–2.170)	0.001	1.602 (1.194–2.150)	0.002	1.599 (1.137–2.247)	0.007
High	Heavy	228	Ref		Ref		Ref	
Low	Heavy	157	1.468 (1.067–2.019)	0.018	1.493 (1.088–2.049)	0.013	1.804 (1.281–2.543)	0.001
Low	Light	185	Ref		Ref		Ref	
High	Heavy	228	1.118 (0.777–1.610)	0.547	1.109 (0.772–1.592)	0.576	1.233 (0.863–1.761)	0.251

Low or high post‐RT Hb levels: post‐RT Hb levels < or ≥12.45 g/dL; Light or heavy smokers: Smoking index < or ≥ 27.5 pack‐years; Ref, reference; HR, hazard ratio; CI: confidence interval.

## Discussion

Our study was designed to assess the predictive value of Hb levels and their interactions with cigarette smoking on prognosis in patients with NPC. Hb levels and smoking habits were carefully extracted from medical records. To improve the accuracy of self‐reported smoking, we excluded the patients whose records differed from those of physicians and nurses. Because all patients had been encouraged to cease smoking after their NPC diagnoses, they might have hidden or minimized their smoking habits. In addition, previous studies showed there were no significant differences in tumor complete response rate or OS between recent quitters and persistent smokers during radiotherapy 19, 20. Although our study only focused on smoking before treatment, the results should be convincing.

In this study, we determined the cutoff points of pre‐RT Hb (14.25 g/dL), mid‐RT Hb (13.06 g/dL), and post‐RT Hb (12.45 g/dL) levels by ROC curve analysis. To review the previous studies 21, 22, 23, 24, 25, 26, the Hb cutoff points associated with poor survival in head and neck cancer ranged from 11.5 to 14.5 g/dL. In addition, Prosnitz et al. elucidated that patients with Hb levels less than 13 g/dL have a higher rate of tumor hypoxia compared with those who have Hb levels of 13 g/dL or higher 2. Taken together, our cutoff values were similar to those for previous results.

Previous research 27, 28 has found that smokers had significantly higher mean Hb levels than did never‐smokers, but no significant differences have been noted between ex‐smokers and never‐smokers, and mean hemoglobin levels increased progressively with the number of cigarettes consumed. We had similar results, but the correlations between Hb levels and smoking status were not significant (*P *>* *0.05) after age, gender, and clinical stage were controlled for. In smokers (excluding the four female patients), our analyses showed that there were also no correlations between pack‐years and Hb levels after age and clinical stage were controlled for, and we speculated about the possible reasons for this. First, the study subjects were different; previous studies used only healthy people, whereas ours used cancer patients. Secondly, previous studies showed that the effects of smoking on Hb levels appeared to be more pronounced in women 27, 28, but the vast majority of smokers (>99%) in our study were male.

It has been confirmed that impaired tumor oxygenation or hypoxia in patients with low Hb levels induces radioresistance, as there is a great deal of indirect evidence from the associations between tumor control and Hb levels 1, 26, 29. However, hemoglobin alone cannot reflect the true oxygen status of the tumor, partly because blood's oxygen unloading capacity can differ widely 30. In smokers, oxygen delivery to tissues is influenced by the formation of carboxyhemoglobin (COHb), and blood COHb levels in nonsmokers are usually 1–2%, whereas smokers can have values as high as 15–20%; in general, however, levels are approximately 4–6% 30, 31. Increased blood COHb levels were found to reduce oxygen supply to tumors and to influence the efficacy of radiation therapy 32. However, previous studies analyzed the effects of Hb levels on survival without considering different smoking statuses or the cumulative effects of cigarette smoking, Therefore, these studies cannot provide information on the potential effects of smoking and Hb levels on survival.

In our study, a multivariate analysis in the entire population was performed that included clinical characteristics, tumor variables, Hb levels, and smoking status. The results showed that only post‐RT Hb level was a significant independent prognostic factor. Pre‐RT and mid‐RT Hb levels and smoking status were not independent prognostic factors for survival. There were no multiplicative interactions between Hb levels and smoking status for survival. Compared with patients with low post‐RT Hb levels, patients with high levels exhibited better OS (HR = 0.797, 95% CI, 0.678–0.936; *P *=* *0.006), FFS (HR = 0.81, 95% CI, 0.692–0.951; *P *=* *0.010), and LR‐FFS (HR = 0.725, 95% CI, 0.608–0.865; *P *<* *0.001). The survival benefit of OS and FFS was mainly derived from improved regional tumor control, and post‐RT Hb level was not associated with distant failure. These results were consistent with those from previous studies 1, 3, 7, 26, 33. It is generally acknowledged that these findings are primarily a consequence of impaired tumor oxygenation or hypoxia, resulting in a more aggressive and radiotherapy‐resistant tumor phenotype 1.

Some studies 1, 2, 3, 7, 26 have uncovered that low Hb levels prior to and during treatment have been associated with poor prognosis in HNSCC patients who were treated with radiotherapy; other studies 3, 25, 33 have shown that post‐RT Hb level was associated with survival. Thus, the optimal time to measure Hb levels has never been established. Our study found that only post‐RT Hb level was a significant independent prognostic factor for survival. Tumor hypoxia may be one reason for this, but it is most likely not the only one; low Hb levels can also be an epiphenomenon of malignant tumor behavior. Malignant tumors can activate the immune and inflammatory systems and produce cytokines, including interferons, TNF, and interleukin‐1, which further inhibit erythropoietin, affect the life span of erythrocytes and impair iron metabolism 1, 34.

The cumulative effect of smoking was also strongly associated with survival in NPC patients. In the cohort of smokers, post‐RT Hb level and pack‐years were found to be significant, independent predictors of prognosis after T stage (T1‐2 vs. T3‐4), N stage (N0‐1 vs. N2‐3), treatment group (RT vs. CRT), and BMI (continuous variable) were adjusted for. There were no multiplicative interactions, but a significant, positive, additive interaction was found between low post‐RT Hb level (<12.35 g/dL) and high pack‐years (≥27.5) on OS (Fig. 2). These results showed and 66.5% of patient deaths could be attributed to the additive interaction between low post‐RT Hb level and high pack‐years. Subsequent stratified analysis showed that heavy smokers with low post‐RT Hb had an HRs of 2.295 (95% CI, 1.684–3.129; *P *<* *0.001) for death and 2.267 (95% CI, 1.887–3.714; *P *<* *0.001) for locoregional recurrence compared with light smokers with high post‐RT Hb level.

The positive additive interaction between low post‐RT Hb level and high pack‐years indicates that the combined effect of low post‐RT Hb level and high pack‐years on survival is larger than the sum of the individual effects of the above two risk factors on survival; in fact, this interaction is synergistic. There are a number of possible explanations for this. First, patients with low Hb levels have decreased oxygen affinity and oxygen‐carrying capacity, whereas heavy smokers have higher carbon monoxide levels, which have an affinity with hemoglobin that is 200–280 times greater than that with oxygen 30. All these factors shift the oxygen affinity curve to the left and cause a decreased diffusion distance of oxygen and a subsequent increase in the percentage of hypoxic cells in a tumor. Furthermore, the vasoconstrictive effect of nicotine in heavy smokers may influence the amount of oxygen that is carried to the tissues 32. In addition, the influence of low Hb levels and smoking on comorbidity, quality of life, the development of secondary cancers, and/or other related health issues has been well established 35, 36, 37, 38. Thus, an interaction between Hb level and smoking could have been anticipated.

Our study has a number of strengths. First, to the best of our knowledge, this is the first study to analyze the interaction between low Hb level and high pack‐years, which will provide relatively accurate risk prediction for NPC patients in the future. Secondly, a relatively large database (*n *=* *2440) was used to reduce the sources of bias and confounding. Lastly, we further carried out a stratified analysis to reveal the interaction between Hb level and pack‐years.

The limitations of our study are related to its retrospective nature and the fact that the data were obtained exclusively from one center. Additionally, the proportion of female smokers in this study was relatively small (only four female smokers), and thus, it remains uncertain whether the same conclusions could be extrapolated to female patients. Thirdly, the chemotherapy modality varied differently, which might have had a confounding effect. In addition, due to the very small percentage of patients who received 3DRT and IMRT, the conclusions should be interpreted with some caution in these patients. Additional molecular biological studies are needed to explore the interior interaction mechanisms in heavy smokers with low post‐RT Hb levels.

In summary, our data suggest that post‐RT Hb is an important predictor of survival in patients with NPC, and a low post‐RT Hb level correlates with poor survival. There is a positive additive interaction between post‐RT Hb level and pack‐years on survival, which means that the combination of low post‐RT Hb level and high pack‐years will result in poorer survival and an increased risk of relapse. Patients who are undergoing radiation therapy for NPC should be encouraged to discontinue smoking and are often warned against the potential adverse effects of low post‐RT Hb levels. Oncologists should devote particular attention to heavy smokers with low post‐RT Hb levels in the future.

## Conflicts of Interest

No potential conflicts of interest were disclosed.

## Supporting information

Figure S1. Flowchart of study design.

Figure S2. Comparison of survival between patients with high and low Mid‐RT Hb levels.

Figure S3. Comparison of survival between patients with high and low Pre‐RT Hb levels.
