# Supplementary figures and images for: The effects of hemoglobin levels and their interactions with cigarette smoking on survival in nasopharyngeal carcinoma patients
Source: Cancer Med. 2016 Jan 28;5(5):816–26. doi: 10.1002/cam4.647 (PMC4864811; doi:10.1002/cam4.647)

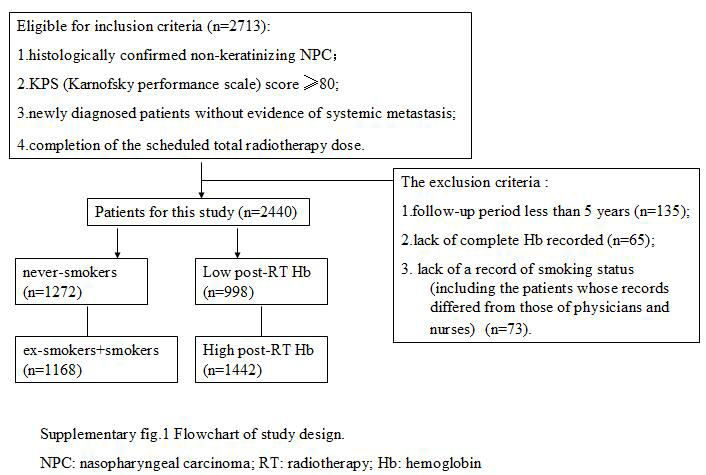

Supplement: Supplementary file 1 — Figure S1. Flowchart of study design. [file CAM4-5-816-s001.tif]

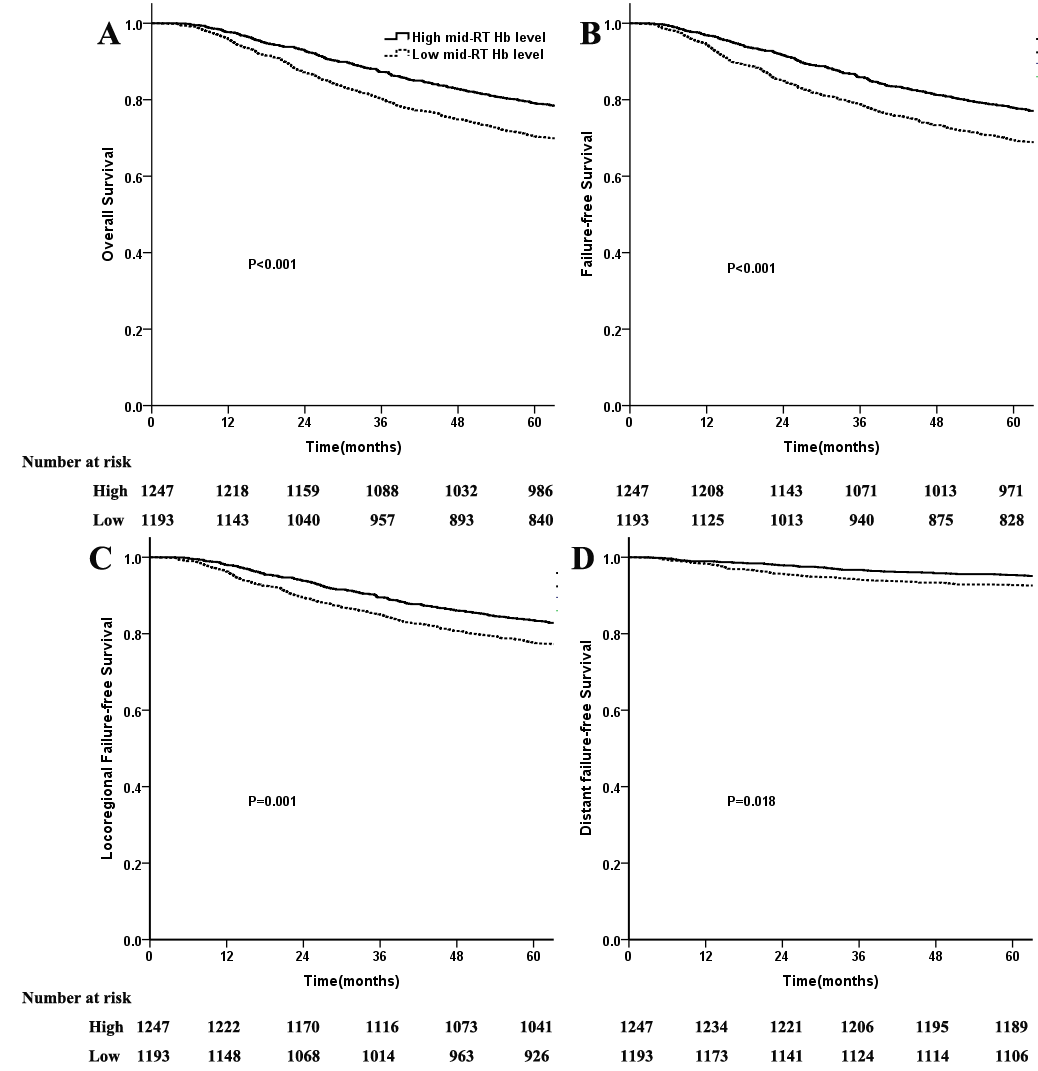

Supplement: Supplementary file 2 — Figure S2. Comparison of survival between patients with high and low Mid‐RT Hb levels. [file CAM4-5-816-s002.tif]

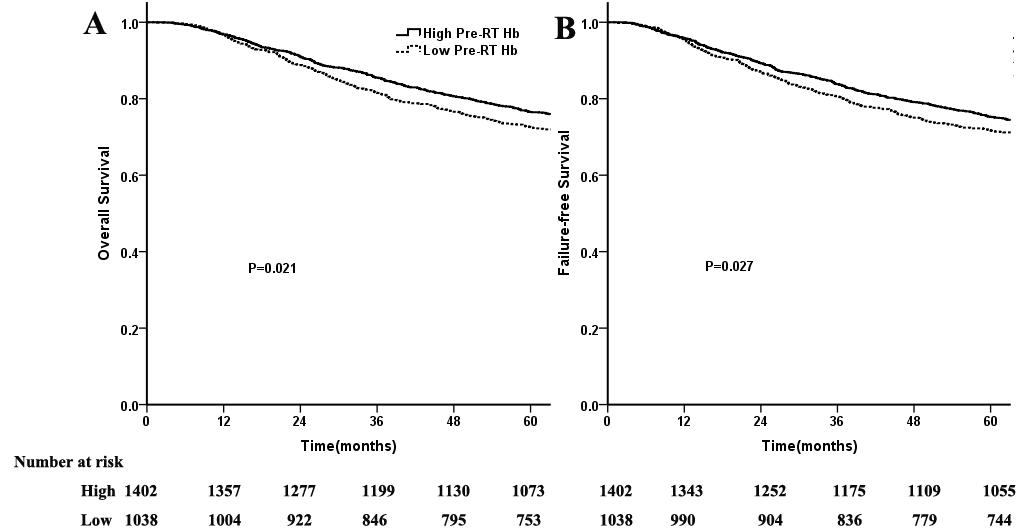

Supplement: Supplementary file 3 — Figure S3. Comparison of survival between patients with high and low Pre‐RT Hb levels. [file CAM4-5-816-s003.tif]
